# Elucidation of the Antiferroelectricity Mechanism in CsBi(MoO_4_)_2_

**DOI:** 10.1021/acsmaterialsau.5c00246

**Published:** 2026-04-15

**Authors:** Andries van Hattem, Gilles Wallez, Nick T. H. ter Veer, Indu Dhiman, Kathy Dardenne, Jörg Rothe, Rudy J. M. Konings, Anna L. Smith

**Affiliations:** † Radiation Science and Technology Department, Faculty of Applied Sciences, 378606 Delft University of Technology, Mekelweg 15, Delft 2629JB, The Netherlands; ‡ 27063 Sorbonne Université, Paris 75005, France; ¶ Radionuclide Speciation Department, Institute for Nuclear Waste Disposal (INE), Karlsruhe Institute of Technology (KIT), Hermann-von-Helmholtz-Platz 1, Eggenstein-Leopoldshafen 76344, Germany

**Keywords:** neutron diffraction, antiferroelectricity, CsBi(MoO_4_)_2_, lone pair, X-ray absorption spectroscopy, thermal expansion

## Abstract

Although the antiferroelectric compound CsBi­(MoO_4_)_2_ has been known for a long time, the underlying mechanism remained poorly understood. No temperature-dependent crystallographic investigation was performed to solve this. In this work, a neutron diffraction study at 150 K was used to solve the crystal structure of the antiferroelectric phase existing between 135 and 330 K. X-ray absorption spectroscopy at room temperature and temperature-dependent diffraction studies between 150 K and the melting point were used to elucidate the mechanism driving the phase transition. The antiferroelectricity mechanism of CsBi­(MoO_4_)_2_ is revealed to be driven by the temperature-dependent Bi displacement caused by the Bi 6*s* ^2^ lone pair. The thermal expansion of CsBi­(MoO_4_)_2_ between 150 K and its melting point is determined, as well. The current work solves the long-standing question of the origin of the antiferroelectricity in CsBi­(MoO_4_)_2_.

## Graphical abstract

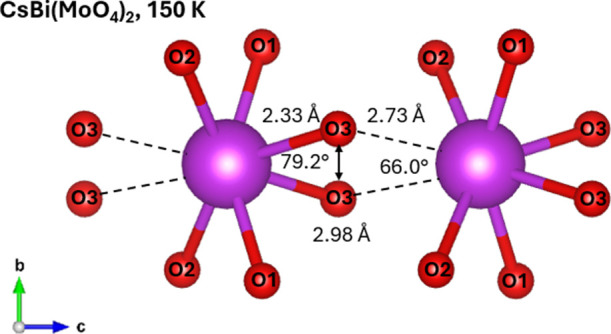

## Introduction

Despite the systematic studies of double molybdates over multiple decades,^1–6^ the double molybdate CsBi­(MoO_4_)_2_ has still some structural mysteries to unravel, both at ambient and at nonambient temperatures. A crystal of CsBi­(MoO_4_)_2_ was first grown by Klevtsov, Vinokurov, and Klevtsova in 1973, who stated that the compound is isostructural to CsPr(MoO_4_)_2_ and crystallizes in space group *Pccm* (*D*_2*h*_^3^, 49).^1,7,8^ They did not observe polymorphic transitions using differential thermal analysis and measured the melting point to be 945 ± 15 K.^7^ Pelikh and Zvyagin, on the other hand, reported two phase transitions in CsBi­(MoO_4_)_2_, observed while studying the temperature-dependent permittivity in a single crystal.^9^ They observed a discontinuity at 135 K as well as an inflection point at 330 K, and they interpreted the low-temperature anomaly as an improper antiferroelectric transition. A few years later, they reported the effect of uniaxial pressure on CsBi­(MoO_4_)_2_ and concluded that the transition at 135 K is accompanied by a remarkable volume change.^10^ In 1980, Brilingas et al.^11^ investigated the dielectric properties of CsBi­(MoO_4_)_2_ crystals at microwave frequencies, observing transitions at the same temperatures. They classified the transition at 135 K as a first-order ferroelastic transition and the transition at 330 K as a second-order displacive-type antiferroelectric transition. Other investigations into the phase transitions of CsBi­(MoO_4_)_2_ report on the sample size effect (Pelikh^12^), the specific heat (Stokka and Samulionis^13^), and vibrational spectra by infrared and Raman spectroscopy (Zvyagin and Kut’ko,^14^ Hanuza and Maczka,^15^ Maczka, Hanuza and co-workers^16–19^ ).

Zvyagin and Kut’ko were the first authors to conjecture that the actual room temperature structure may have a lattice parameter corresponding to a double unit cell, and they proposed that the crystal structure evolves according to $D_{2h}^{3}\overset{330K}{\to }D_{2h}^{8}\overset{135K}{\to }C_{i}^{1}$ .^14^ Similar to the later spectroscopic studies of Maczka, Hanuza and several co-workers,^15–19^ the physics of the compound was interpreted in analogy to other complex molybdate compounds. Although the $D_{2h}^{3}\overset{330K}{\to }D_{2h}^{8}$ transition will turn out to be the proper assignment, it is not caused by the molybdate moiety but by the Bi­(III) 6*s* ^2^ lone pair activity. Important to note is that the spectroscopic studies were interpreted with isolated molybdate tetrahedra. Moreover, Maczka et al. claimed that at the second-order transition, the point group symmetry for the molybdate ions changes from *C*_*s*_ to *C*_1_, and the phase transition seems to be of a displacive nature.^16^ The doubling of the unit cell via doubling of the *a* lattice parameter is hypothesized by them to be caused by an antiphase displacement of the Cs^+^ ions or a [Bi­(MoO_4_)_2_]^−^-layer. In a later article, they reject this hypothesis and propose a monoclinic space group, even though they rejected the monoclinic option themselves earlier based on the available diffraction data.^16,18^ In their latest article (2005), Maczka, Hanuza, and co-workers wrote that room temperature has an unknown symmetry. In this high-pressure Raman study, two phases next to the antiferroelectric and paraelectric phases were found.^19^

X-ray diffraction (XRD) or neutron diffraction (ND) studies at nonambient pressure or temperature have not been reported to date, and according to several of the discussed studies, the original space group assigned to the room temperature phase of CsBi(MoO_4_)_2_ is incorrect, given its physical properties. In this article, we describe the antiferroelectric (135 < *T* < 330 K) structure of CsBi­(MoO_4_)_2_ as determined using XRD and ND at various temperatures, together with the phase transition mechanism at 330 K. The analysis of the crystal structure of CsBi­(MoO_4_)_2_ is supported by X-ray absorption spectroscopy (XAS) at the Mo K-edge. The ND, especially at low temperature, is key to measuring the displacive phase transition reported below. The XAS spectroscopy confirms the local Mo environment, in line with expectations based on older literature.

## Experimental Section

### Synthesis

CsBi­(MoO_4_)_2_ was synthesized using a two-stage solid-state synthesis process. All samples were handled in Ar-filled gloveboxes. First, Cs_2_MoO_4_ was synthesized from Cs_2_CO_3_ (99.99%, Alfa Aesar) and MoO_3_ (99.5%, Alfa Aesar). The precursors were mixed in stoichiometric quantities, ground thoroughly, and heated twice for 12h at 973 K in an alumina crucible under an oxygen atmosphere. The sample was reground intermittently. Second, Bi_2_(MoO_4_)_3_ was synthesized from Bi_2_O_3_ (99.99%, Sigma-Aldrich) and MoO_3_ at 875 K for 12 h under oxygen flow twice with intermittent regrinding. Finally, CsBi­(MoO_4_)_2_ was synthesized by mixing Cs_2_MoO_4_ and Bi_2_(MoO_4_)_3_. After thorough grinding, the mixtures were heated in an alumina crucible for 12 h at 750 K. After cooling, the mixture was reground and again heated for 12 h at 750 K. No unaccounted Bragg reflections were found. Alternatively, Cs_2_CO_3_ (99.9%, Sigma-Aldrich), MoO_3_ (99+ %, Alfa Aesar), and Bi_2_O_3_ (99+ %, Prolabo) were directly mixed in the 1:4:1 molar ratio. The reagents were slowly heated to 573 K, pressed into a pellet, and reheated at 873 K. Only faint impurity peaks were found on the diffraction pattern.

### X-ray Diffraction

X-ray diffraction studies were performed at room temperature using laboratory and synchrotron X-ray radiation. For the high-temperature studies (298–873 K), a Panalytical X’Pert Pro diffractometer was used in Bragg–Brentano geometry (*U* = 45 kV, *I* = 40 mA) with Cu *K*_α1_ radiation, a Ge(111) fore monochromator, and a PIXcel detector equipped with an Anton Paar furnace. Synchrotron diffraction measurements were performed at ID22 at the ESRF in Grenoble.^20^ Data analysis was performed using the profile refinement method^21,22^ in the FullProf suite.^23^ The synchrotron diffraction data were refined using the Thompson-Cox-Hastings function with spherical harmonics expansion.

### Neutron Diffraction

ND was measured at the PEARL beamline at the Hoger Onderwijs Reactor (HOR) at TU Delft.^24^ The sample was encapsulated in a vanadium Null-alloy can. For the measurements at room temperature and below, the can was hermetically closed with an In-seal. For the measurement at 423 K, a Cu O-ring was used as the seal. The data were collected with a fixed wavelength of 0.1667 nm (Ge(533) plane) in the angle range 11° ≤ 2θ ≤ 159°. Data analysis was performed using the profile refinement method^21,22^ in the FullProf suite.^23^ Thompson-Cox-Hastings functions were used to describe the peak profile.

### X-ray Absorption Spectroscopy

XAS samples were prepared by pressing pellets of 8 mm diameter consisting of a mixture of the compound and boron nitride (BN) powder. These pellets were enclosed in a Kapton foil. X-ray absorption spectroscopy measurements were performed at the INE Beamline of the KIT light source (Karlsruhe, Germany) with 2.5 GeV and 150–170 mA as operating conditions in the Karlsruhe Research Accelerator (KARA) storage ring.^25^

The beamline was a Ge(422) double-crystal monochromator (DCM). Rh-coated mirrors before (flat, cylindrically bent) and after (toroidal) the DCM are used to collimate and focus the synchrotron beam, respectively, producing a spot size of 300 μm by 500 μm at the sample surface. Transmission and fluorescence geometries could be measured in unison. Samples were probed around the K-edge of the Mo (20 keV). In the X-ray absorption near-edge structure (XANES) spectrum, the absorption edge energy threshold position *E*_0_ was determined using the inflection point of the spectrum. The inflection point was determined using the zero crossing of the second derivative and verified by the maximum of the first derivative. The prepeak position was selected as the peak maximum via the first derivative. Each spectrum was aligned using the XANES spectrum of a metallic Mo reference foil. The ATHENA software was used for analysis.^26^

The extended X-ray absorption fine structure (EXAFS) part of the spectrum was recorded at up to 16 Å^–1^. The Fourier transform was taken using the Hanning window over the *k*-range 3.0–11.5 Å^–1^ (d*k* = 2). Subsequently, the ARTEMIS software was used to fit the EXAFS data.^26^ The phases and amplitudes for the interatomic scattering paths were calculated with the software FEFF8.40. The shift in threshold energy Δ*E*_0_ = −1.4 eV, while the amplitude for all paths *S*_0_^2^ = 0.83. The distance resolution is given by Δ*R* = π/(2·Δ*k*), which is equal to 0.18 Å. The model obtained after refinement of the ND data at 298 K was used as a starting point in the optimization (Tables 1 and 3). The first coordination shell and the high-amplitude scattering paths of the second coordination shell were optimized. The Mo–O1, Mo–O2, and Mo–O4 scattering paths were merged, while the Mo–O3 scattering path was kept independently.

**Table 1:** Cell Parameters of CsBi­(MoO_4_)_2_ ^a^

technique	space group	temp. (K)	a (Å)	b (Å)	c (Å)	volume (Å^3^)
ND	*Pccb*	150	5.1592(2)	18.6361(7)	8.1790(3)	786.4
ND	*Pccb*	200	5.1528(3)	18.6999(9)	8.1909(4)	789.3
ND	*Pccb*	298	5.1211(2)	18.9272(6)	8.2265(3)	797.4
ND	*Pccm*	423	5.1297(4)	9.5210(7)	8.2574(6)	403.3
sXRD	*Pccb*	298	5.12536(1)	18.98122(3)	8.23987(1)	801.7
XRD	*Pccb*	298	5.12985(3)	18.90333(11)	8.21747(5)	796.9
XRD^7^	*Pccm*	298	5.14(3)	9.44(1)	8.20(9)	397.9

a Uncertainties are given by FullProf. sXRD is synchrotron X-ray diffraction.

## Results and Discussion

A yellow powder was obtained after the synthesis that seemed pure on laboratory XRD patterns. However, as explained in the introduction, the crystallography and the antiferroelectric behavior are conflicting. To elucidate the phase transition mechanism, it was found necessary to probe all atoms and to probe them at nonambient temperatures. This section is organized as follows: First, the ND results at 150 K will be presented. Second, the diffraction and XAS results at 298 K are discussed. Third, the thermal expansion based on diffraction results at nonambient temperature is presented. Finally, an elucidation of the phase transition mechanism at 330 K is given.

### Toward the Crystal Structure of the Antiferroelectric Phase: Refinement against the ND Data at 150 K

Using the earlier proposed crystallographic model for CsBi(MoO_4_)_2_ in space group *Pccm* (49),^7^ a first series of refinements against the XRD data were performed to determine the thermal expansion of CsBi­(MoO_4_)_2_. A nonlinearity in cell parameters when crossing 330 K was observed. However, expected atomic shifts for a second-order phase transition of displacive nature between 298 and 330 K are expected to be very small, stimulating us to collect ND data at a temperature where the displacement should be most pronounced, i.e., at around 150 K. For the initial refinement to the synchrotron X-ray diffraction, the model in space group *Pccm* (49) was taken as well. In the Fourier difference map, the Bi atom was found to split between two symmetric positions, which hinted at a lone pair effect, but the extent of the effect was quite small to conclusively assign it to a doubled cell.

The space group search was initiated based on the following criteria:

1. The symmetry should remain orthorhombic according to the absence of peak splitting or broadening of the synchrotron diffraction pattern recorded at room temperature.
2. The space group should be a subgroup of that of the high-temperature form owing to the second-order character of the transition.
3. A polar space group was hypothesized, considering the dielectric anomaly^11^ as a hint for a ferroelectric to paraelectric phenomenon.

The *Pccm* space group is centrosymmetric and thus in contradiction with a dielectric effect, but it is a proper description for the paraelectric phase (*T* > 330 K).

The interpretation of the ND at 150 K was based on the following arguments. As previous research suggested,^14^ the peak of the dielectric permittivity at 330 K implies a doubled cell parameter or superstructure. This led us to consider the three *mm*2-type subgroups of *Pccm*, namely, *P*2*cm*, Pc2*m*, and *Pcc*2 with unchanged axis orientation. In the *Pccm* phase, Cs and Bi occupy independent 222-positions, implying doubling of the *b*-parameter, which induces the loss of half of these special positions in the *P*2*cm* and *Pcc*2 forms. In these symmetries, only one of Cs or Bi can remain on a special position (2.. and..2), while in Pc2*m*, both can keep occupying a 0.2. position. The five most likely options were tested (*P*2*cm* with Cs in 0.2. or Bi in 2.. or Cs and Bi in 0.2.; *Pcc*2 with Cs in..2 or Bi in..2) against the ND pattern obtained at 150 K using a split pseudo-Voigt peaks profile function with Caglioti polynomial width. Independent isotropic atomic displacement parameters (ADPs) were refined for all atoms, while the background was modeled by interpolation of selected points that were refined.

The refinement of the five tested models led to an unambiguous outcome: four of them resulted in unrealistic MoO_4_ tetrahedra deformations, thus requiring distance constraints in order to comply with spectroscopic interpretation of molybdate tetrahedra in earlier studies and our XANES results presented *supra*. Distance constraints still yielded unrealistic models, with highly divergent O–Mo–O angles, high or negative ADPs, and high *R*_Bragg_ factors. On the opposite, the model in space group *Pcc*2 (27) with Bi in..2 was satisfactory in every aspect, i.e., positive definite ADPs, regularly shaped MoO_4_-tetrahedra without constraints. However, the so-obtained atomic positions turned out to match the centric *Pccb* symmetry (space group 54). This means that the condition of a polar space group required by a ferroelectric phase was too excessive, and the interpretation of CsBi(MoO_4_)_2_ to be antiferroelectric between 135 and 330 K by Brilingas et al. to be correct.^11^

Refinement of the model against ND data obtained at 150 K in space group *Pccb* is shown in Figure 1, while the cell parameters are given in Table 1. The choice of space group *Pccb* allowed a drastic reduction of the number of variable atomic coordinates (17 in *Pccb* instead of 34 in *Pcc*2), whereas the fit quality remains reasonable with only a slight degradation. In view of parsimony, this *Pccb* symmetry was preferred in the following. The geometry of the MoO_4_-tetrahedrons and the ADPs are satisfactory. The refined parameters are listed in Table 2. The Bi shift, as found on the Fourier plot at 150 K, is shown in Figure 2. The crystal structure at 150 K is shown in Figure 3. The ‘empty spaces’ next to the Bi atoms are interpreted as an expression of a Bi­(III) lone pair, see Figure 4. The cumulated bond valence values using Brese and O’Keeffe’s model^27^ are 1.05 for Cs­(I), 3.21 for Bi­(III), and 5.44 for Mo­(VI). The four different Mo–O distances (see Table 5) are in line with the statement of Maczka et al.^16^ that the molybdate tetrahedra at room temperature should have *C*_1_ point group symmetry.

**Figure 1:**
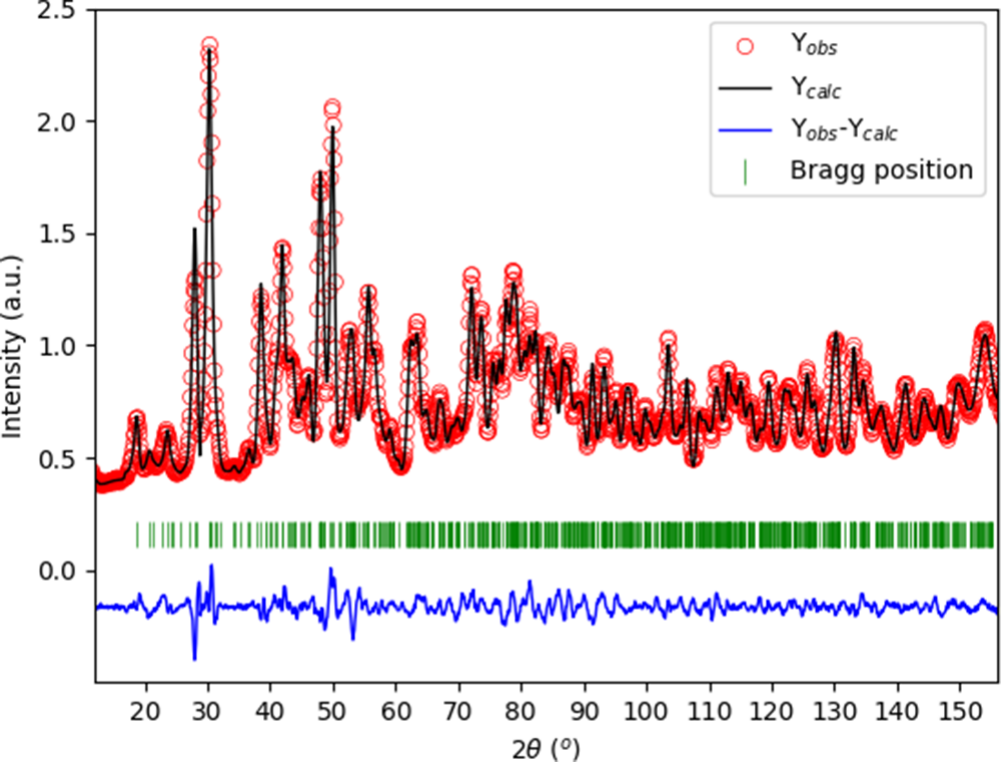
Experimental (*Y*_obs_, in red) and calculated (*Y*_calc_, in black) neutron diffraction patterns at 150 K. The difference between calculated and experimental intensities *Y*_calc_ – *Y*_obs_ is shown in blue. The angular positions of Bragg reflections are shown in green. Measurement at λ = 1.667 Å.

**Figure 2:**
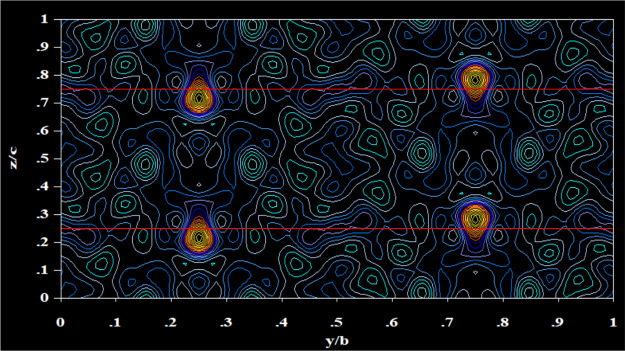
Fourier plot at 150 K, showing the most pronounced Bi shift from its ideal position. The archetype position coincides with the drawn red lines at *z* = 0.25 and *z* = 0.75.

**Figure 3:**
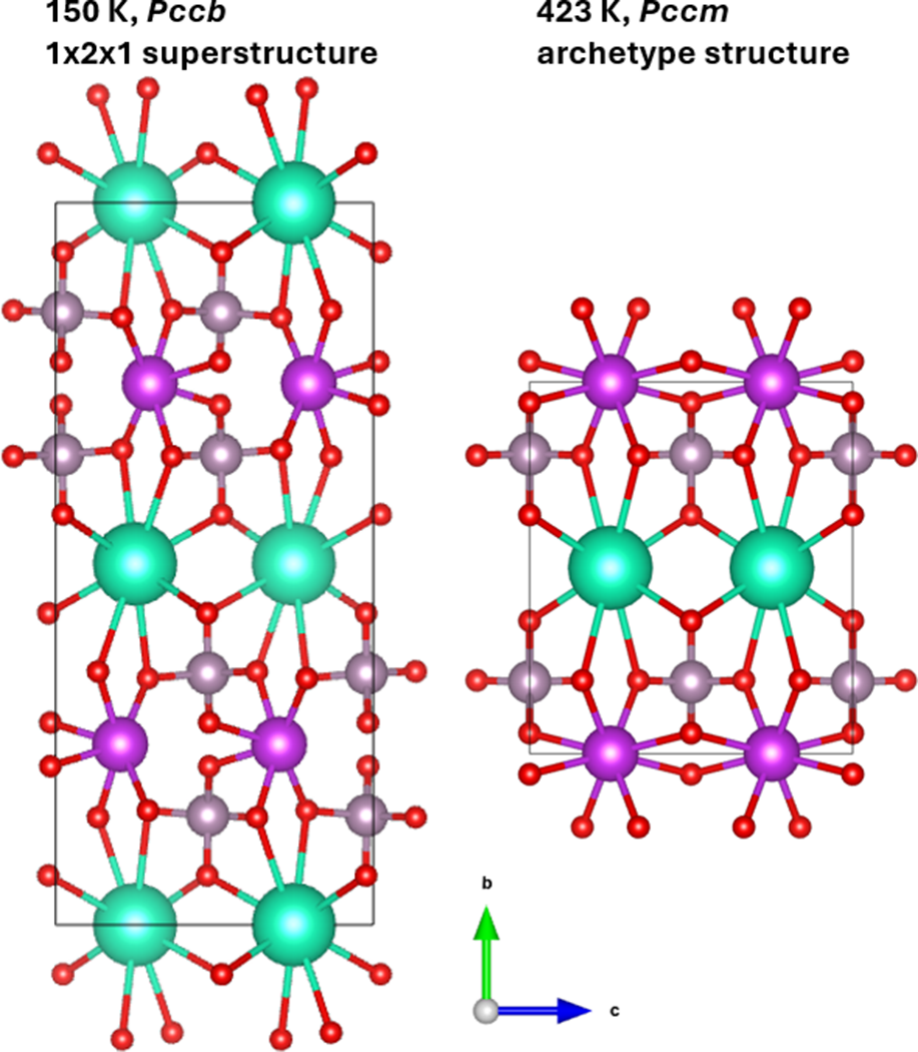
*Pccb* superstructure of antiferroelectric CsBi­(MoO_4_)_2_ (stable between 135 and 330 K) compared to the high-temperature archetype *Pccm* structure (stable from 330 K up to the melting point). The aquamarine, purple, gray, and red colors stand for Cs, Bi, Mo, and O atoms, respectively.

**Figure 4:**
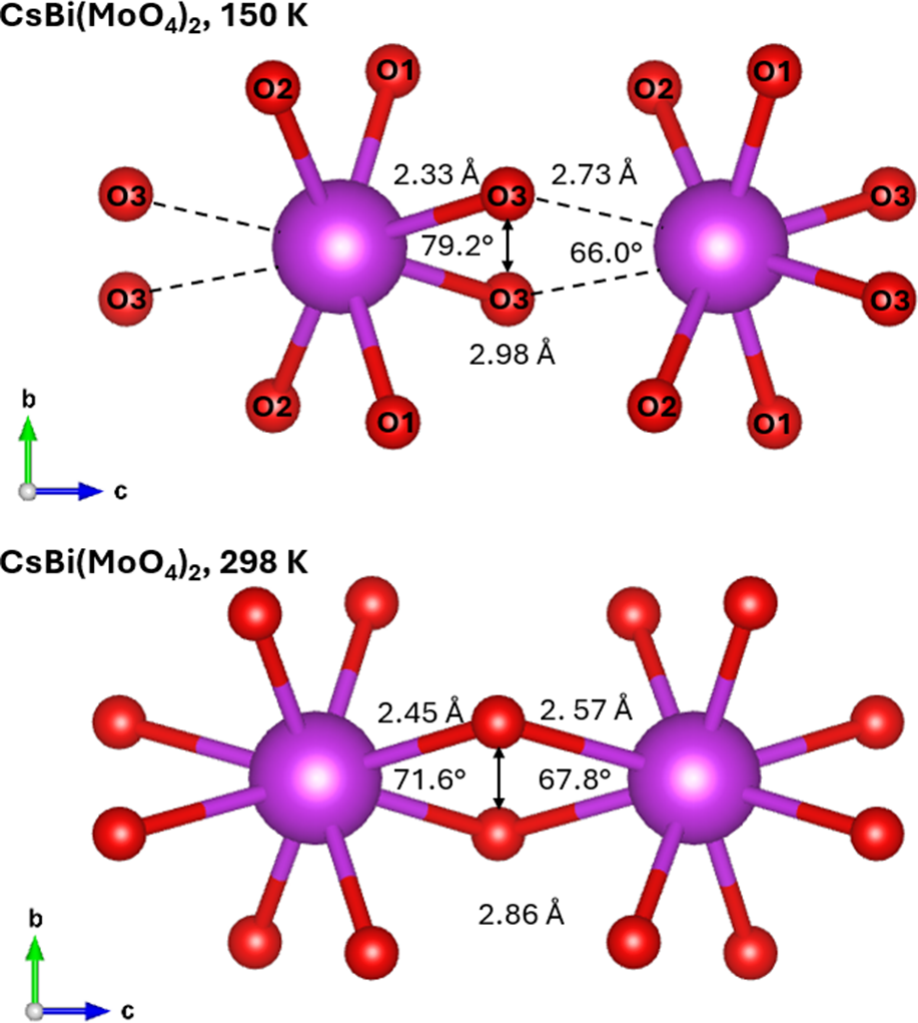
Close view of the Bi coordination at 150 and 298 K, showing that the lone-pair effect is much more pronounced at lower temperatures.

**Table 2:** Refined Atomic Positions and Isotropic Atomic Displacement Parameters Based on Neutron Diffraction Data at 150 K in Space Group *Pccb* (54)^a^

atom	Wyckoff	x/a	y/b	z/c	B (Å^2^)
Bi	4d	0	0.25	0.2020(6)	0.33(6)
Cs	4c	0.040(19)	0.5	0.25	1.66(13)
Mo	8f	0.5209(8)	0.34863(19)	–0.0206(5)	0.60(5)
O1	8f	0.7760(9)	0.3510(3)	0.1317(6)	1.39(9)
O2	8f	0.6965(8)	0.3415(2)	–0.2112(6)	0.99(7)
O3	8f	0.2669(7)	0.2802(2)	–0.0160(6)	0.46(7)
O4	8f	0.3853(8)	0.4316(2)	–0.0203(7)	0.76(7)

a *R*_p_ = 7.29, *R*_wp_ = 8.85, *R*_exp_ = 2.21, χ^2^ = 16.0.

### Crystal Structure of CsBi­(MoO_4_)_2_ at Room Temperature

Profile refinement was applied against the ND pattern obtained at 298 K and the synchrotron diffraction pattern at 298 K as well, as shown in Figures 5 and 6, respectively. Cell parameters at various temperatures are listed in Table 1. The optimized atomic parameters are presented in Tables 3 and 4. In Figure 4, the change in the Bi–O bond distances is highlighted. Toward the phase transition temperature (330 K), the O3 atoms move to a more intermediate position. The relation to the phase transition will be elaborated on in infra. Here, it highlights the difficulty of probing the atomic-scale structure of CsBi­(MoO_4_)_2_ close to the phase transition temperature.

**Table 3:** Refined Atomic Positions and Isotropic Atomic Displacement Parameters Based on Neutron Diffraction Data at 298 ± 5 K in Space Group *Pccb* (54)^a^

atom	Wyckoff	x/a	y/b	z/c	B (Å^2^)
Bi	4d	0	0.25	0.2313(7)	1.60(6)
Cs	4c	0.000(3)	0.5	0.25	1.80(9)
Mo	8f	0.5259(5)	0.34856(13)	–0.0090(6)	1.56(4)
O1	8f	0.7445(11)	0.3501(3)	0.1569(5)	2.04(9)
O2	8f	0.7241(11)	0.3440(3)	–0.1889(5)	1.74(9)
O3	8f	0.2532(5)	0.2820(16)	–0.0100(7)	1.40(5)
O4	8f	0.3745(6)	0.4310(16)	–0.0164(7)	2.18(8)

a *R*_p_ = 5.74, *R*_wp_ = 6.73, *R*_exp_ = 2.70, χ^2^ = 6.20.

**Table 4:** Refined Atomic Positions and Isotropic Atomic Displacement Parameters Based on Synchrotron Diffraction Data at 298 K in Space Group *Pccb* (54)^a^

atom	Wyckoff	x/a	y/b	z/c	B (Å^2^)
Bi	4d	0	0.25	0.2461(2)	1.360(8)
Cs	4c	0.0082(6)	0.5	0.25	3.025(19)
Mo	8f	0.5179(2)	0.34935(3)	–0.0005(3)	1.242(15)
O1	8f	0.7266(17)	0.3472(5)	0.1476(9)	0.7(2)
O2	8f	0.7359(15)	0.3401(5)	–0.1885(9)	0.03(17)
O3	8f	0.2812(8)	0.2818(3)	–0.002(2)	0.42(10)
O4	8f	0.3665(8)	0.4231(3)	0.001(2)	0.71(11)

a *R*_p_ = 11.9, *R*_wp_ = 13.9, *R*_exp_ = 2.43, χ^2^ = 32.7.

**Figure 5:**
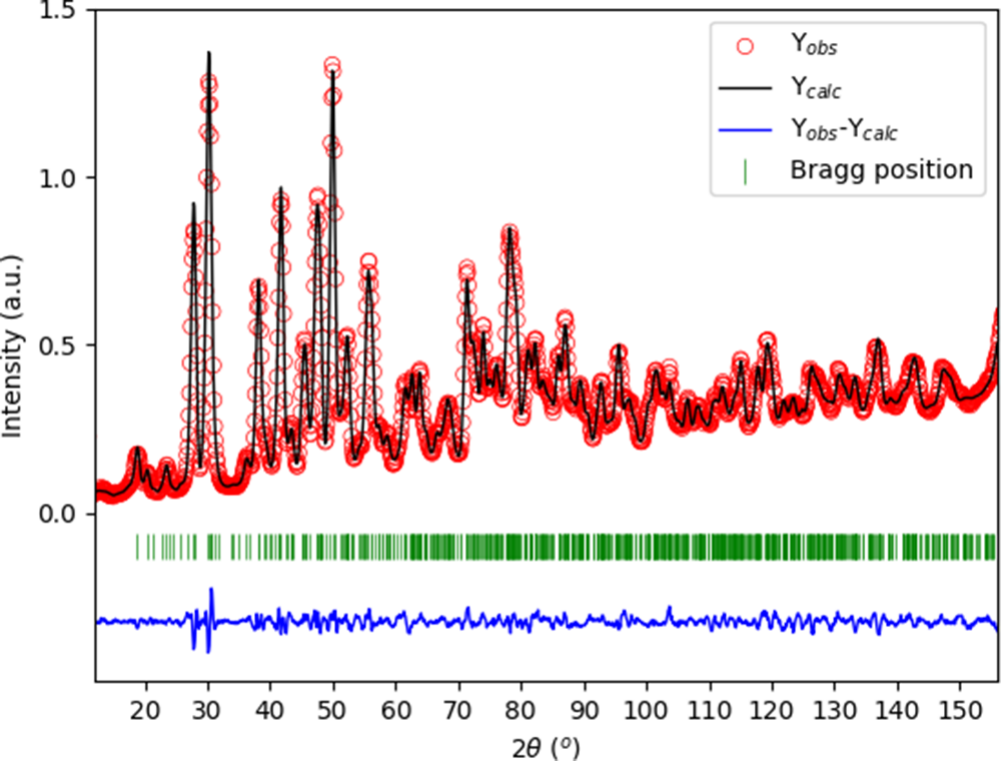
Experimental (*Y*_obs_, in red) and calculated (*Y*_calc_, in black) neutron diffraction patterns at 298 K. The difference between calculated and experimental intensities *Y*_calc_ – *Y*_obs_ is shown in blue. The angular positions of Bragg reflections are shown in green. Measurement at λ = 1.667 Å.

**Figure 6:**
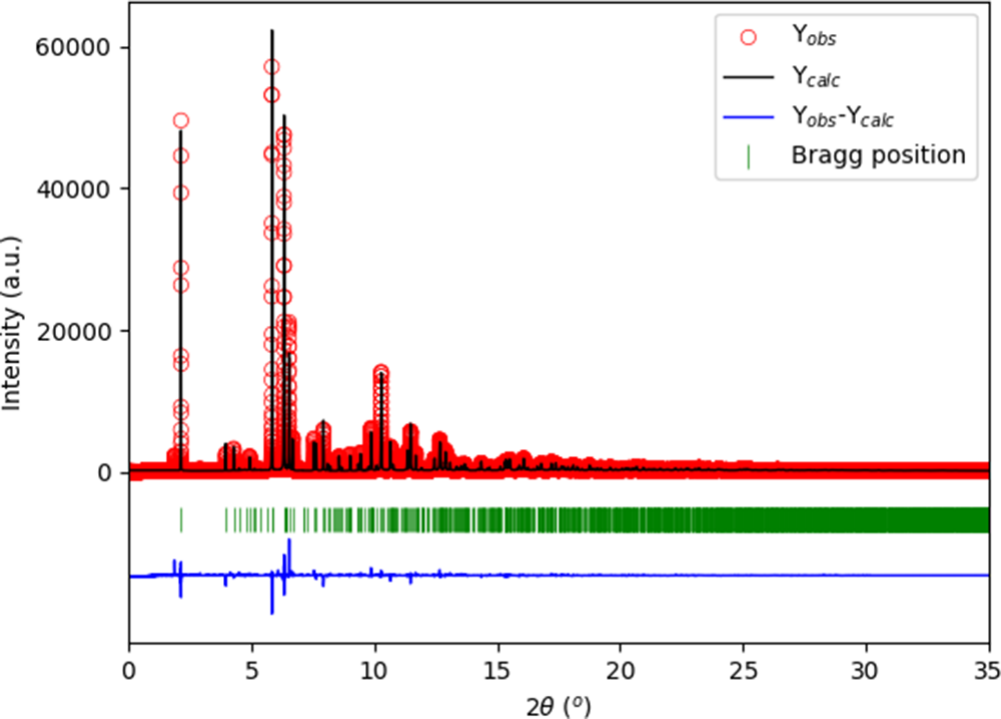
Experimental (*Y*_obs_, in red) and calculated (*Y*_calc_, in black) synchrotron diffraction patterns at 298 K. The difference between calculated and experimental intensities *Y*_calc_ – *Y*_obs_ is shown in blue. The angular positions of Bragg reflections are shown in green. Measurement was at λ = 0.35451 Å.

X-ray absorption spectroscopy at the Mo K-edge was performed at room temperature in an early stage of this work as well, and the data was interpreted with the herein newly proposed structural model.

The XANES spectrum is shown in Figure 7. The edge position of CsBi­(MoO_4_)_2_ aligns with that of MoO_3_, confirming the expected hexavalent oxidation state of Mo. The pre-edge feature in the CsBi­(MoO_4_)_2_ spectrum is characteristic for tetrahedral MoO_4_^2–^ ions, as octahedral, distorted octahedral, and tetrahedral Mo-surroundings give rise to clearly distinguishable pre-edge peaks.^28–30^

**Figure 7:**
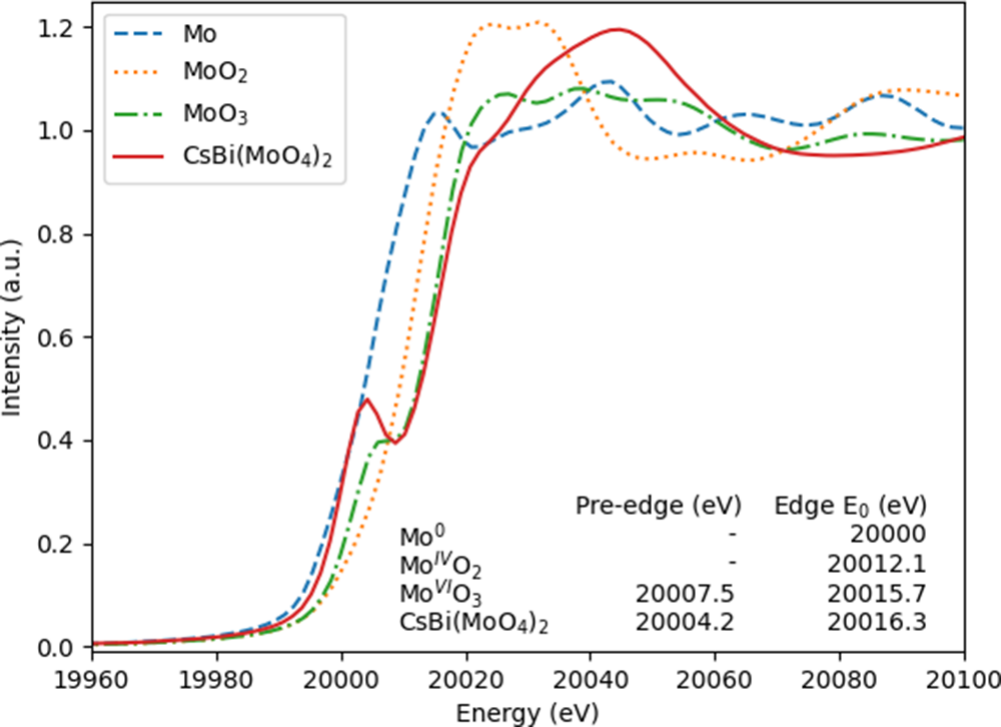
X-ray absorption near edge structure spectrum obtained on CsBi­(MoO_4_)_2_ and Mo, MoO_2_, and MoO_3_ reference materials at room temperature.

The EXAFS spectrum at the Mo K-edge was fit to the crystal structure with a doubled cell parameter, and four independent oxygen positions were obtained in this work (Tables 1 and 3). The fits of the data to this model are shown in the *r*-space and *k*-space in Figure 8. The refined Mo–O distances are listed in Table 5. The MoO_4_-tetrahedron was optimized in the EXAFS fit with three equivalent bond distances and one slightly elongated distance. Although the difference is not significant when judged from the possible spatial resolution of the measurement, the found elongation seems to have a physical explanation, as the longer Mo–O distance is related to the O3 atom, which is the atom that is connecting the Bi atoms; see Figure 4. At 298 K, just below the phase transition temperature, O3 is only slightly repelled by the lone pair of one of the Bi atoms and attracted by the other Bi atom. The Debye–Waller factor of this Mo–O3 scattering path is also an order of magnitude larger than the other Mo–O scattering paths in the first coordination shell. Thus, although the mechanism in CsBi­(MoO_4_)_2_ is driven by the Bi^3+^ lone pair effect (vide infra*)*, it affects the Mo–O distances as well, though a strict conclusion cannot be drawn based on the EXAFS data obtained herein, as the difference in distance is statistically insignificant. Temperature-dependent XAS studies would be valuable to get further insights, or existing temperature-dependent vibrational spectroscopy data can be reinterpreted with the full structural model obtained in this work (e.g.,^16^ and later studies).

**Figure 8:**
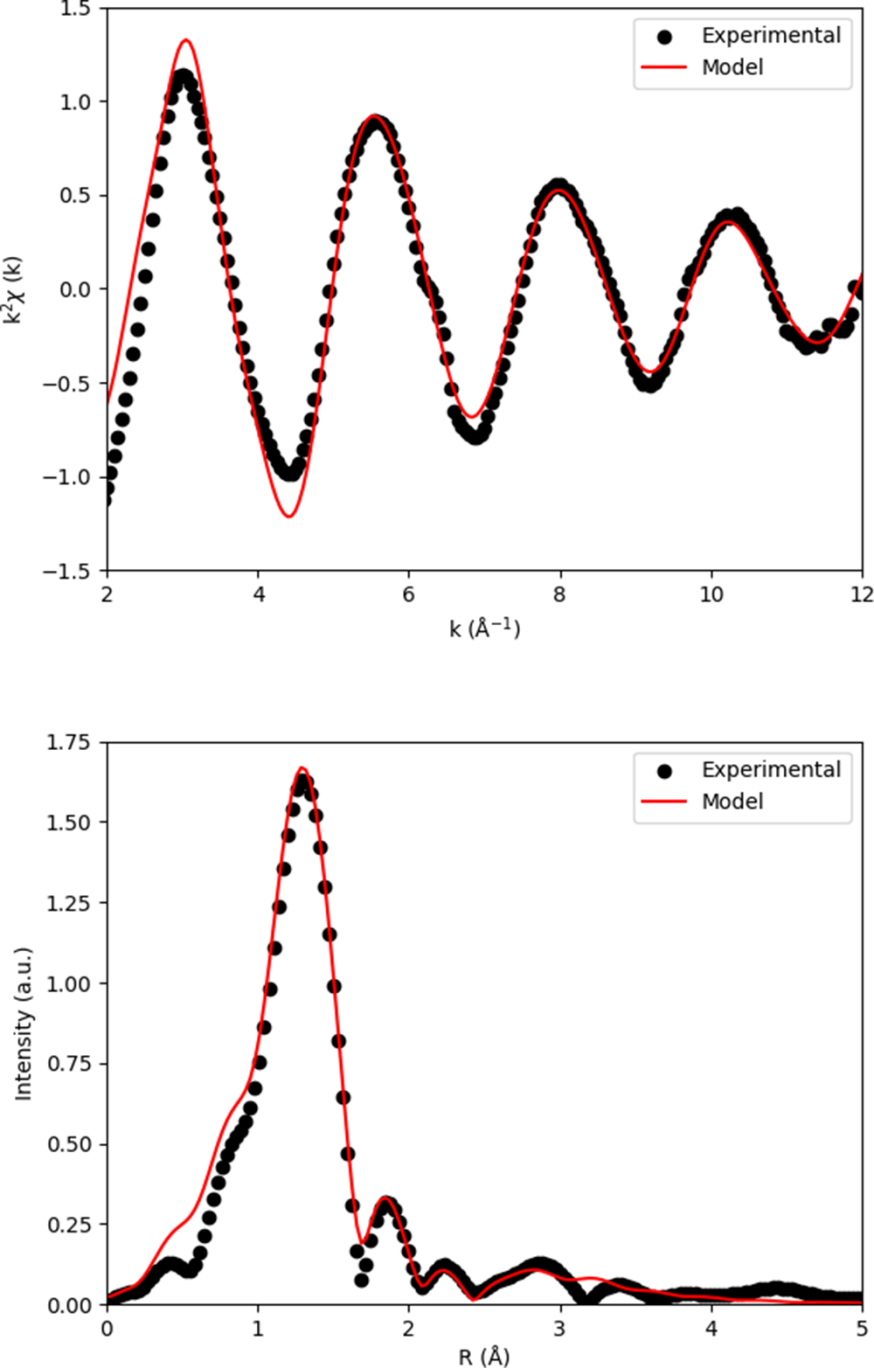
Extracted experimental (black dots) and fitted (red line) EXAFS signal of CsBi­(MoO_4_)_2_ at room temperature.

**Table 5:** Bond Lengths and Debye-Waller Factors Obtained from the Fit to the EXAFS Data^a^

scattering pathway		CN	distance EXAFS (Å)	Debye–Waller factor (Å^2^)	distance ND 298 K (Å)
Mo	O1	3 × 1	1.78	0.0016	1.766(7)
	O2				1.797(7)
	O4				1.743(5)
Mo	O3	1	1.87	0.0112	1.881(4)
Mo	O2(2)	1	3.01	0.0016	2.929(7)
	O3(2)	1	2.85	0.0067	2.717(4)
Mo	O1(2)	1	3.12	0.0016	3.078(7)

a Δ*E*_0_ = −1.4 eV. The *R*-factor for this data set equals 1.1%. The multiple-scattering path of the first Mo–O1­(O2/O4) was also added to the model.

## Temperature-Dependence of Crystal Properties

Profile refinement was applied against the ND pattern obtained at 200 K by using the newly obtained model. The result is shown in Figure 9, with cell parameters reported in Table 1. The optimized atomic parameters are presented in Table 6.

**Table 6:** Refined Atomic Positions and Isotropic Atomic Displacement Parameters Based on Neutron Diffraction Data at 200 K in Space Group *Pccb* (54)^a^

atom	Wyckoff	x/a	y/b	z/c	B (Å^2^)
Bi	4d	0	0.25	0.2051(7)	0.31(9)
Cs	4c	0.025(3)	0.5	0.25	2.0(2)
Mo	8f	0.521(10)	0.3477(3)	–0.0195(7)	0.75(9)
O1	8f	0.7708(12)	0.3519(4)	0.1373(8)	1.30(12)
O2	8f	0.7039(11)	0.3424(4)	–0.2061(7)	0.97(13)
O3	8f	0.2685(11)	0.2810(3)	–0.0149(9)	0.92(12)
O4	8f	0.3853(11)	0.4316(4)	–0.01592(10)	1.28(11)

a *R*_p_ = 9.16, *R*_wp_ = 10.8, *R*_exp_ = 2.43, χ^2^ = 19.8.

**Figure 9:**
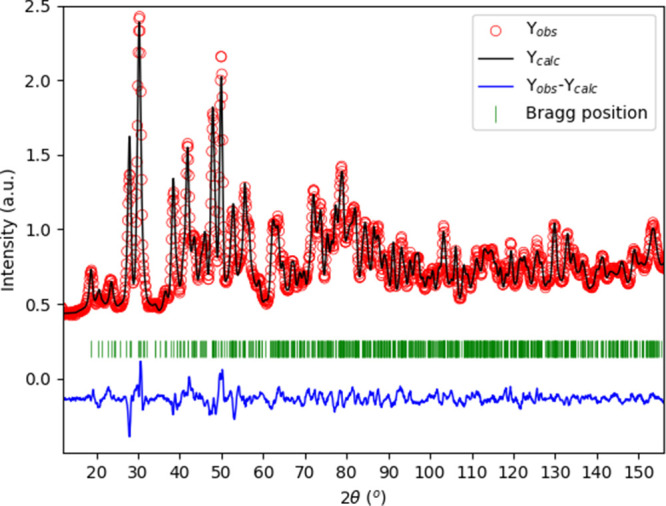
Experimental (*Y*_obs_, in red) and calculated (*Y*_calc_, in black) neutron diffraction patterns at 200 K. The difference between calculated and experimental intensities *Y*_calc_ – *Y*_obs_ is shown in blue. The angular positions of Bragg reflections are shown in green. Measurement at λ = 1.667 Å.

Also, profile refinement was applied against the ND pattern obtained at 423 K using the archetype model. The result is shown in Figure 10. The cell parameters are again reported in Table 1, and the optimized atomic parameters are presented in Table 7.

**Table 7:** Refined Atomic Positions and Isotropic Atomic Displacement Parameters Based on Neutron Diffraction Data at 423 K in Space Group *Pccm* (49)^a^

atom	Wyckoff	x/a	y/b	z/c	B (Å^2^)
Bi	2e	0	0	0.25	1.08(11)
Cs	2g	0	0.5	0.25	1.8(2)
Mo	4q	0.5478(13)	0.1931(7)	0	0.89(10)
O1	8r	0.2663(8)	0.1973(5)	0.3365(5)	0.68(7)
O2	4q	0.2420(16)	0.0553(8)	0	1.65(15)
O3	4q	0.3461(17)	0.3575(8)	0	1.65(16)

a *R*_p_ = 13.7, *R*_wp_ = 15.2, *R*_exp_ = 5.76, χ^2^ = 6.99.

**Figure 10:**
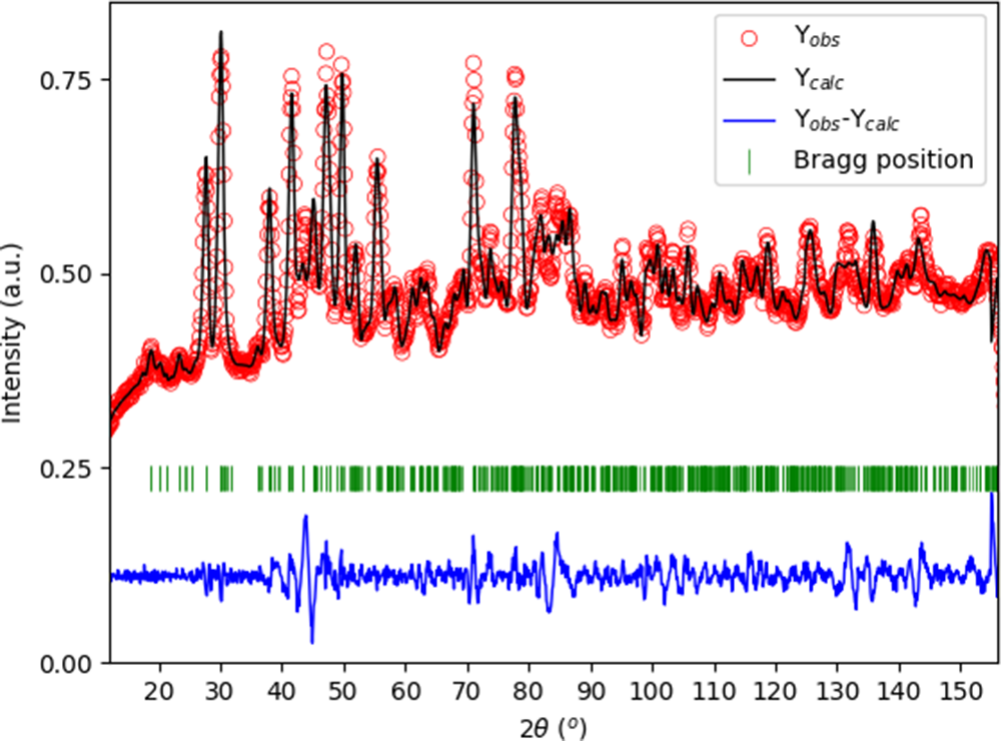
Experimental (*Y*_obs_, in red) and calculated (*Y*_calc_, in black) neutron diffraction patterns at 423 K. The difference between calculated and experimental intensities *Y*_calc_ – *Y*_obs_ is shown in blue. The angular positions of Bragg reflections are shown in green. Measurement at λ = 1.667 Å.

High-temperature XRD data (*T* > 330 K) were optimized against the archetype *Pccm* model. The relation between the *Pccb* superstructure at 150 K and the *Pccm* archetype structure at 423 K is visualized in Figure 3.

The cell parameters obtained using ND were combined with those obtained by high-temperature X-ray diffraction measurement. The *b*-axis was rescaled to the archetype (high temperature) phase, and the resulting cell volume with increasing temperature is shown in Figure 11. The resulting volumetric thermal expansion α_V_ = 87 × 10^–6^ K^–1^ using all data from both X-ray and neutron diffraction. Effectively, CsBi­(MoO_4_)_2_ shows a continuous volumetric expansion over the phase transition around 330 K, which is in agreement with a second-order transition.

**Figure 11:**
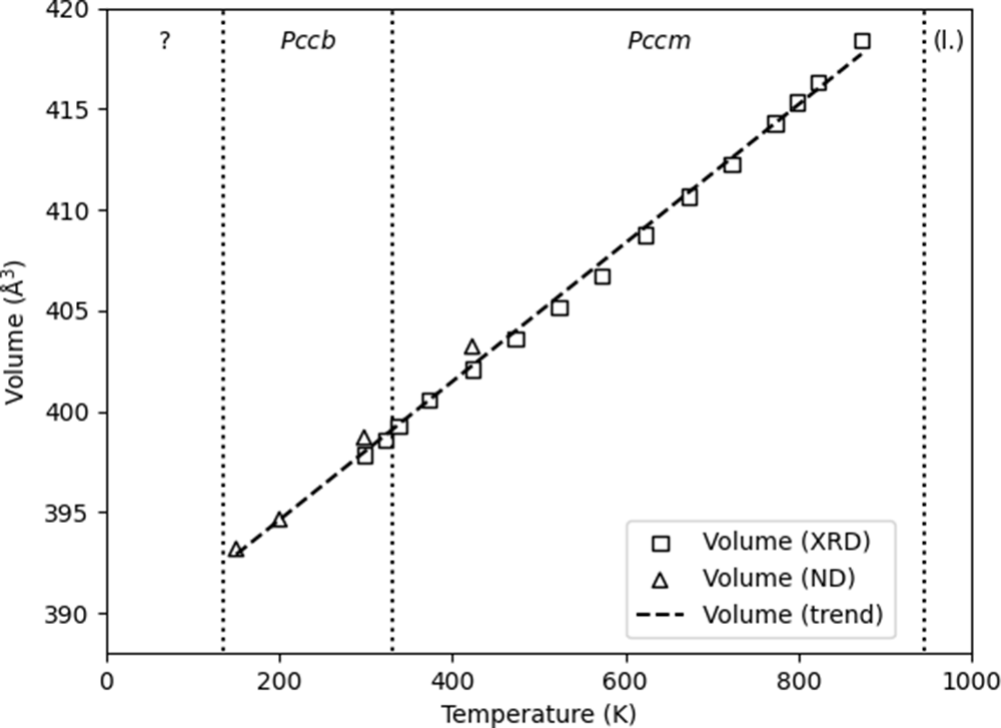
Change of unit cell volume, as rescaled to the archetype structure. The dotted lines indicate the phase transition temperatures of CsBi­(MoO_4_)_2_. The dashed line is a guide for the eye.

The cell parameters obtained from neutron and X-ray diffraction were all rescaled to the archetype structure, and the relative expansion compared to room temperature was calculated; the resulting relative thermal expansion along the three axes is shown in Figure 12. A change of expansion appears near the temperature of the anomaly observed by dielectric measurements (330 K).^11^ This confirms the existence of a second-order phase transition. The phenomenon is particularly visible on the *a* = *f*(*T*) and *b* = *f*(*T*) plots, and to a lesser extent on the *c* = *f*(*T*) plot. The strong contraction along *a* below the Curie temperature becomes much smaller above the Curie temperature. The *a*-axis contracts, because of the additional bonds between MoO_4_ and Bi, as the O atom is less repelled by the Bi 6*s* ^2^ lone pair with temperature increasing from 150 K to the Curie temperature.

**Figure 12:**
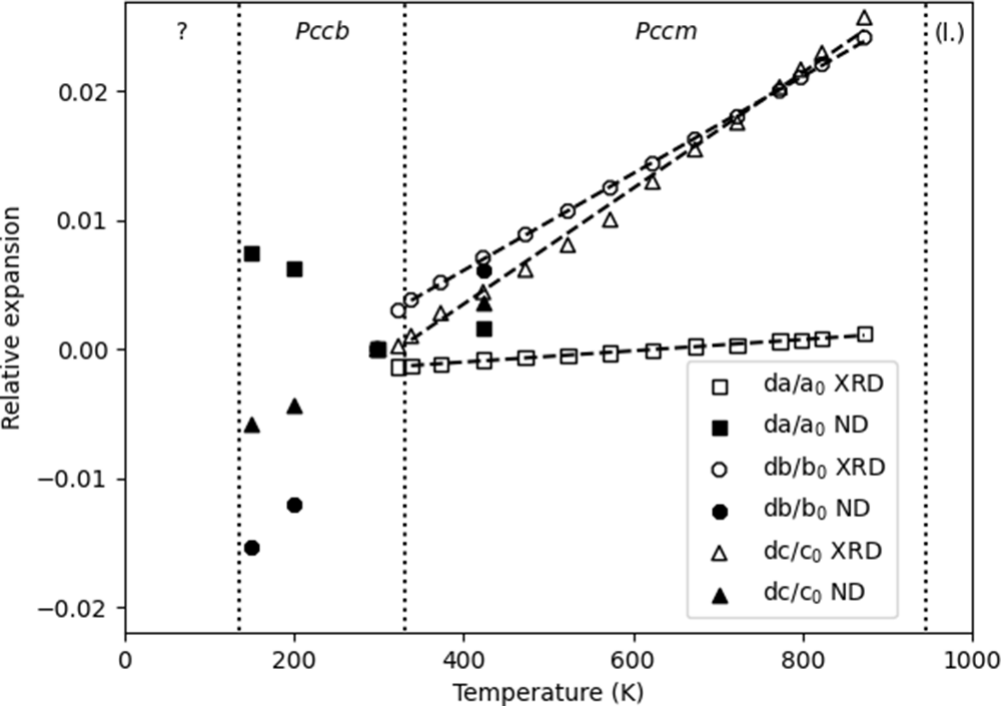
Relative thermal expansion following the *a*, *b*, and *c* axes as measured by low-temperature neutron and high-temperature X-ray diffraction. The dotted lines indicate the phase transition temperatures of CsBi­(MoO_4_)_2_. The dashed lines are guides for the eye.

Beyond the Curie temperature, the coefficients of thermal expansion are α_*a*_ = 4.4 × 10^–6^ K^–1^, α_*b*_ = 38 × 10^–6^ K^–1^, and α_*c*_ = 45 × 10^–6^ K^–1^ for the temperature range 338–873 K. The strong expansion along *b* can be ascribed to the weakness of the Cs–O bonds, while the strong expansion along *c* is also due to repulsive Coulombic interactions between edge-sharing Bi–O polyhedra. The moderate expansion following *a* is a consequence of the latter mechanism that induces a contraction of the shared O–O edges.

### Antiferroelectric-Paraelectric Transition Mechanism

The crystal structure at 150 K as shown in Figure 3 reveals the mechanism of the antiferroelectric-paraelectric phase transition at 330 K: First, the antiferroelectric phase exhibits a highly asymmetric environment around Bi­(III), with a ‘umbrella-shaped’ coordination polyhedron due to the stereochemical activity of the 6*s* ^2^ lone pair: the Bi–O distances range from 2.33 Å (opposite) to 2.74 Å (in front), see Figure 4. This configuration is probably responsible for the apparent discrepancy between the expected and calculated bond valences of Bi­(III). A similar excess in bond valence sum is observed in isoelectronic structures such as α-PbO.^31^ Second, the doubling of the *b*-parameter appears to be driven by the alternation of the Bi 6*s* ^2^-dipoles following the *b*-axis. The alternation of these opposite-oriented dipoles (and to a lesser extent, of those formed by the more regular CsO_8_ and MoO_4_ polyhedra) makes the structure an antiferroelectric array, a rather common property among oxides containing cations with lone pairs.^32–34^

To assess whether the Bi displacement is the driving force of the second-order phase transition, a phenomenological Landau model has been developed. As an order parameter, the Bidisplacement is calculated via its *z*-coordinate, *i.e., z*_displacement_ = 0.25 – *z*(*T*) using the values in Tables 2, 6, and 3. The Curie-temperature and constant factor are optimized such that the model follows $\sqrt{\left(T_{C}-T\right)}$ , yielding *T*_C_ = 323.2 K. The result is shown in Figure 13. The optimized *T*_C_ value is in line with literature values of 325 and 330 K.^9,13^

**Figure 13:**
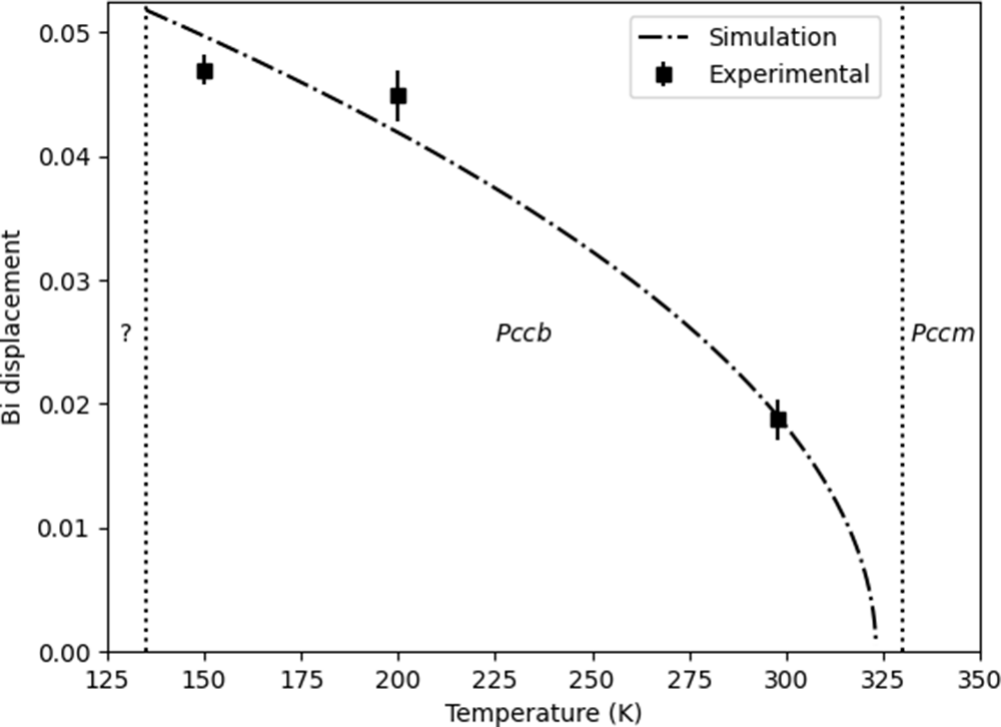
Landau model using the Bi displacement as the order parameter. *T*_C_ = 323.2 K. The dotted lines indicate literature values of 135 and 330 K for the phase transitions, respectively. The error bars are the standard deviations of FullProf multiplied by the score of Bérar and Lennan.^35^

With the herein obtained diffraction data, the earlier conjecture of *D*_2h_^8^ symmetry is proven correct,^14^ although the cell is obtained by a doubling of the *b*-parameter and the mechanism is not primarily driven by the molybdate-tetrahedra but by the Bi^3+^6*s* ^2^ lone pair, which is affecting the molybdate tetrahedra.

## Conclusions

The role of the Bi^3+^ 6*s* ^2^ lone pairs has been neglected for the interpretation of the antiferroelectric phase of CsBi­(MoO_4_)_2_. The crystal structure of CsBi­(MoO_4_)_2_ has herein been investigated using neutron and X-ray diffraction in the temperature range of 150–873 K. XANES spectroscopy at room temperature confirmed the presence of MoO_4_^2–^ ions and the hexavalent oxidation state of Mo. The mechanism of the antiferroelectric to paraelectric state has been derived from the comparison of the structures obtained by ND at *T* = 150 K and *T* = 298 K. The 6*s* ^2^ lone pairs of the Bi atoms form an antiferroelectric arrangement at low and ambient temperature, and via a displacive mechanism, the archetype symmetry is stable above 330 K. The Mo K-edge EXAFS data at 298 K were fitted to the new model, and a satisfactory fit was obtained, hinting at a minor deformation of the molybdate ion caused by the Bi^3+^ lone pair effect as well.
